# *In Situ* Dendritic Cell Recruitment and T Cell Activation for Cancer Immunotherapy

**DOI:** 10.3389/fphar.2022.954955

**Published:** 2022-08-23

**Authors:** Joonsu Han, Rimsha Bhatta, Yusheng Liu, Yang Bo, Hua Wang

**Affiliations:** ^1^ Department of Materials Science and Engineering, University of Illinois at Urbana-Champaign, Urbana, IL, United States; ^2^ Cancer Center at Illinois (CCIL), Urbana, IL, United States; ^3^ Department of Bioengineering, University of Illinois at Urbana-Champaign, Urbana, IL, United States; ^4^ Carle College of Medicine, University of Illinois at Urbana-Champaign, Urbana, IL, United States; ^5^ Beckman Institute for Advanced Science and Technology, University of Illinois at Urbana-Champaign, Urbana, IL, United States; ^6^ Materials Research Laboratory, University of Illinois at Urbana-Champaign, Urbana, IL, United States; ^7^ Institute for Genomic Biology, University of Illinois at Urbana-Champaign, Urbana, IL, United States

**Keywords:** cancer immunotherapy, solid tumor, hydrogel, dendritic cell, T cell

## Abstract

Cancer immunotherapy has shifted the paradigm for cancer treatment in the past decade, but new immunotherapies enabling the effective treatment of solid tumors are still greatly demanded. Here we report a pore-forming hydrogel-based immunotherapy that enables simultaneous recruitment of dendritic cells and *in situ* activation of T cells, for reshaping the immunosuppressive tumor microenvironment and amplifying cytotoxic T lymphocyte response. The injectable pore-forming hydrogel composed of porogen-dispersed alginate network can form a macroporous structure upon injection into mice, and enables controlled release of granulocyte-macrophage colony-stimulating factor (GM-CSF), a chemoattractant for recruiting dendritic cells, and epacadostat, an inhibitor of indoleamine 2, 3-dioxygenase for activating T cells. We show that gels loaded with GM-CSF and epacadostat, after peritumoral injection, can recruit massive dendritic cells *in situ* and activate effector T cells in the tumor tissues, resulting in enhanced frequency and activation status of dendritic cells, reduced numbers of regulatory T (Treg) cells, and increased CD8^+^/Treg ratios in the tumor microenvironment. This hydrogel-based immunotherapy holds great promise for treating poorly-immunogenic solid tumors.

## Introduction

Cancer immunotherapy has achieved significant progress for clinical cancer treatment in the past decade, especially with the success of checkpoint blockades and adoptive T cell therapies (Lesterhuis et al., 2011; Mellman et al., 2011; Couzin-Frankel, 2013). However, the limited patient response to checkpoint blockades, the poor therapeutic efficacy of T cell therapies against solid tumors, and severe side effects in both have limited their wide applications (Makkouk and Weiner, 2015; Hegde and Chen, 2020; Martin et al., 2020). Among all the factors that undermine the therapeutic benefit of existing immunotherapies against solid tumors, the highly immunosuppressive tumor microenvironment, as characterized by reduced numbers and impaired function of effector T cells and antigen-presenting cells [e.g., dendritic cells (DCs)], remains a key hurdle (Huang et al., 2012; Wang and Mooney, 2018; Phuengkham et al., 2019). Various strategies have been attempted to facilitate the tumor infiltration and activation of effector T cells, inhibit regulatory T (Treg) cells, repolarize tumor-associated macrophages to inflammatory phenotypes, or improve the antigen presentation capability of tumor-resident DCs (Huang et al., 2012; Feng et al., 2018; Donlon et al., 2021; Yang et al., 2021). For example, the combination of chemotherapy or radiation therapy with checkpoint blockades to improve the generation of antigen-presenting DCs and tumor-specific effector T cells have been actively pursued in preclinical studies and clinical trials (Heinhuis et al., 2019; Galluzzi et al., 2020). Hydrogel-based immunotherapies have also been developed to enable controlled release of adjuvants, cytokines, macrophage-depleting/repolarizing agents, or immue checkpoint blockades into the tumor tissues, which were shown to improve the systemic effector T cell responses and overall antitumor efficacy (Wang et al., 2017; Wang et al., 2018; Li et al., 2021). The combination of *in situ* hydrogel-based therapies with systemically administered immunomodulatory agents such as checkpoint blockades was also explored for amplifying the therapeutic efficacy against poorly-immmunogenic solid tumors (Hu et al., 2021; Li et al., 2022).

Immune cell-homing macroporous materials enable the recruitment and modulation of DCs for *in situ* production of antigen-presenting DCs and subsequent priming of antigen-specific T cells, which has demonstrated success in treating melanoma, glioblastoma, colon cancer, and lung cancer, among others (Ali et al., 2009a; Kim and Mooney, 2011; Li and Mooney, 2013; Wang et al., 2020a). In these designs, chemokines such as granulocyte-macrophage colony-stimulating factor (GM-CSF) are gradually released from the implanted or injected macroporous materials to attract immature DCs from the other part of the body. The recruited DCs are then modulated in a pool of antigens and adjuvants within the material, yielding mature antigen-presenting DCs *in situ* (Ali et al., 2009b; Ali et al., 2013; Li et al., 2018). DC-homing macroporous materials have also been utilized to develop *in situ* cancer vaccines where chemotherapeutics were released to induce immunogenic death of tumor cells and massive DCs were recruited *in situ* to process antigens released by dying tumor cells (Wang et al., 2020b). However, the cytotoxicity of chemotherapeutics loaded in the macroporous materials towards the recruited DCs limits the therapeutic potential of the *in situ* cancer vaccine. We envision the combination of DC recruitment and T cell activation, by co-loading chemokines and benign T cell modulators in macroporous materials would enable the improved modulation of the immunosuppressive tumor microenvironment.

The metabolic intervention of T cells, *via* inhibition of certain metabolic pathways related to the suppression of T cell activity, in the tumor microenvironment has emerged as a promising approach to potentiate antitumor immunity (Le Bourgeois et al., 2018; Hao et al., 2020; Guo et al., 2021). These metabolism inhibitors are often less toxic than cytokines conventionally used for T cell activation. For example, the inhibition of indoleamine 2, 3-dioxygenase (IDO1), an enzyme that catalyzes the degradation of tryptophan and can suppress the function of effector T cells, was able to activate CD8^+^ T cells and suppress the proliferation of regulatory T (Treg) cells (Prendergast et al., 2017; Cheong et al., 2018; Ladomersky et al., 2018). Epacadostat, a small molecule IDO1 inhibitor, has been extensively explored for the treatment of various types of cancers (Van den Eynde et al., 2020; Yue et al., 2017; CD80, 2015). However, the poor pharmacokinetics and tumor accumulation of the small-molecule metabolism inhibitors inevitably limit their use for targeted modulation of T cells in solid tumors (Kristeleit et al., 2017; Chen et al., 2019). Biomaterial carriers of these metabolism inhibitors, though, would enable improved delivery to the tumor site, controlled release within the tumor tissues, facilitated metabolic intervention, and thus amplification of the immunomodulatory effect. (Wang and Mooney, 2018; Wang et al., 2017; Weber and Mulé, 2015; Gu and Mooney, 2016)

**FIGURE 1:**
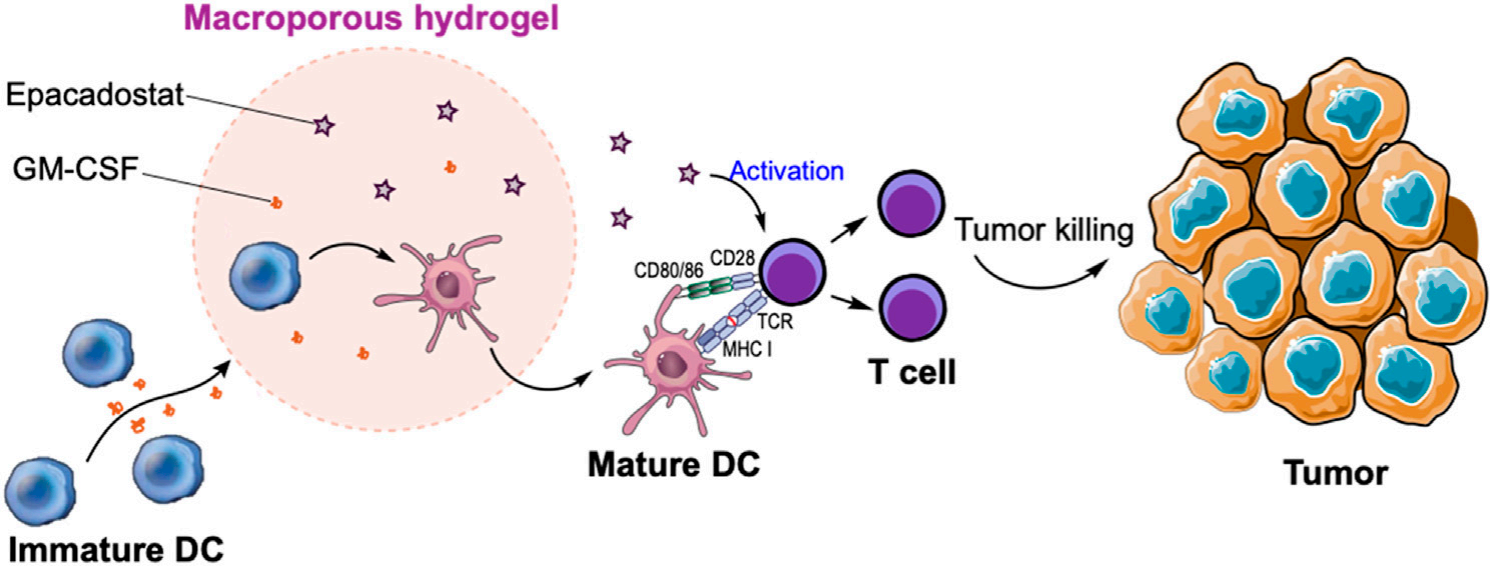
Schematic illustration of *in situ* recruitment of DCs and activation of T cells *via* macroporous hydrogels loaded with GM-CSF and epacadostat. GM-CSF can recruit massive DCs while epacadostat can activate CD8^+^ T cells, resulting in the improved killing of tumor cells.

Here we report the development of a pore-forming hydrogel-based system for simultaneous recruitment of DCs and metabolic intervention of T cells *in situ*, in order to reshape the immunosuppressive microenvironment of solid tumors such as triple negative breast cancers (TNBCs) (Figure 1). The pore-forming gel composed of an alginate network with dispersed porogens can be injected via needles, and form a macroporous structure upon the degradation of porogens at 37°C (Wang et al., 2020a; Wang et al., 2020b). After injection into mice, the formed macroporous gel will enable controlled release of GM-CSF for the recruitment of massive DCs and epacadostat for the activation of effector T cells *in situ*, potentially resulting in the improved modulation of the tumor microenvironment and overall CTL response.

## Results and Discussion

### Synthesis and Characterization of Pore-Forming Gels

**FIGURE 2:**
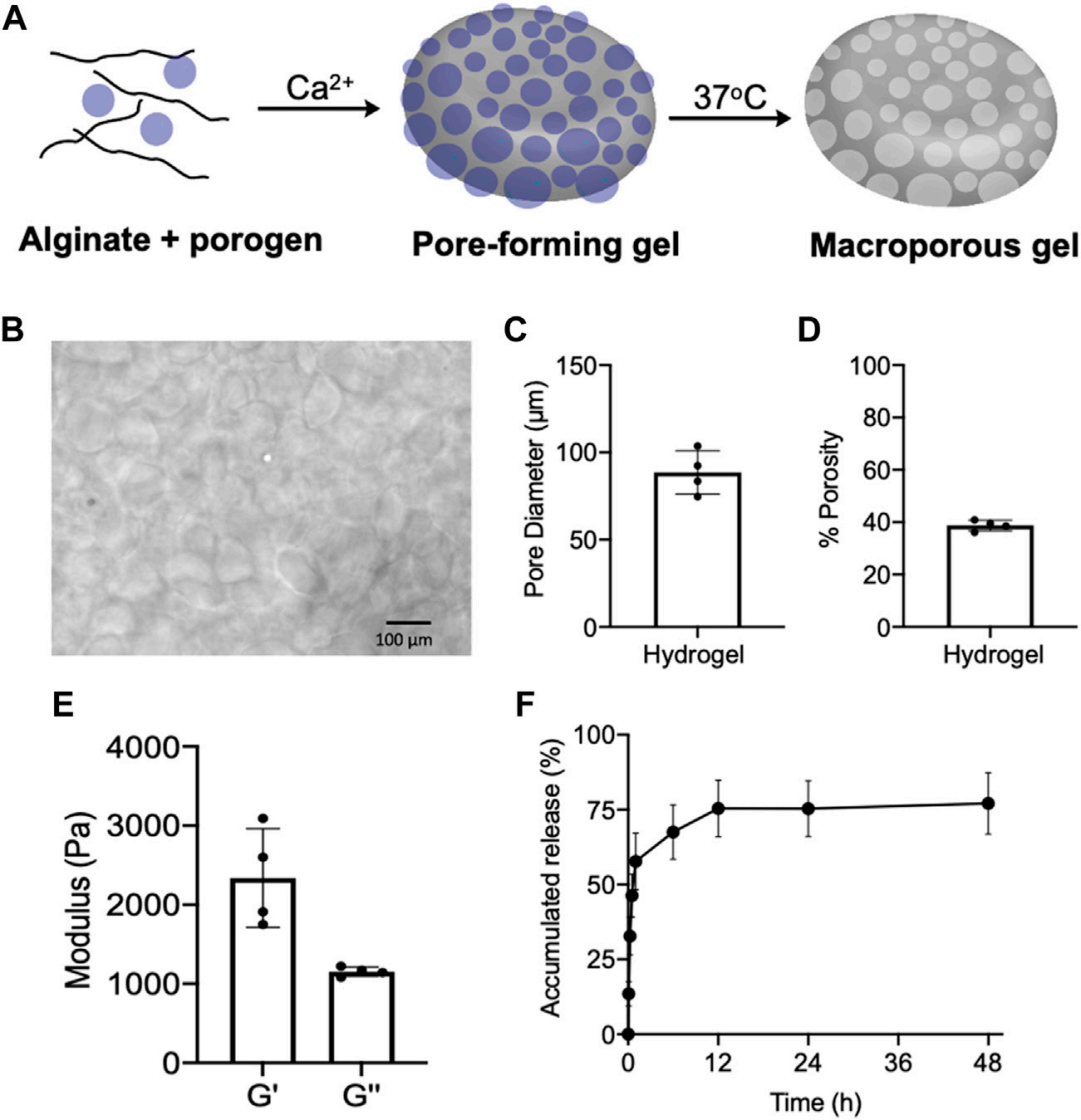
**(A)** Schematic illustration for the fabrication and degradation of pore-forming gels. **(B)** Microscopic images of formed macroporous gels after immersing pore-forming gels in PBS at 37°C for 6 h. **(C)** Pore diameter of formed macroporous gels (*n* = 4). **(D)** Porosity of formed macroporous gels (*n* = 4). **(E)** Storage modulus (G′) and loss modulus (G″) of pore-forming gels (*n* = 4). **(F)** Release kinetics of epacadostat from pore forming gels (*n* = 4). All the numerical data are presented as mean ± SD.

Prior to the fabrication of pore-forming gels, we first synthesized degradable porogen beads via dropwise addition of a mixture of unmodified alginate and oxidized/reduced alginate (1/8, w/w) to a crosslinking bath of CaCl_2_ (Verbeke and Mooney, 2015; Verbeke et al., 2017). The degradable oxidized/reduced alginate was synthesized by first oxidizing alginate with sodium periodate and then reducing the formed aldehyde groups into hydroxyl groups using sodium borohydride. Due to the increased hydrophilicity and reduced steric hindrance of oxidized/reduced alginate, the porogen can be rapidly degraded in aqueous solutions at 37°C. The fabricated porogens were then mixed with unmodified alginate, and further crosslinked by calcium ions to form a pore-forming alginate gel with dispersed porogens (Figure 2A). By immersing the pore-forming alginate gel in PBS at 37°C, macropores with an average diameter of 88.6 µm were formed within the gel network, as confirmed under the microscope (Figures 2B,C). The pore size is large enough to home immune cells (e.g., DCs) which have a diameter of ∼10–15 µm. The resultant macroporous hydrogel has a porosity of ∼40%, as determined by the gel wicking assay (Figure 2D). We also measured the mechanical property of pore-forming alginate gels, which have a storage modulus (G′) of 2,338 Pa and a loss modulus (G″) of 1,153 Pa (Figure 2E). Epacadostat can be easily loaded into the pore-forming alginate gel by co-dissolving it with alginate prior to Ca^2+^ mediated cross-linking. The loaded epacadostat and GM-CSF showed a burst release from the gel within the first 6 h, with the remaining cargo being gradually released over days (Figure 2F; Supplementary Figure S1).

### Epacadostat-Mediated Activation of CD8^+^ T cells

**FIGURE 3:**
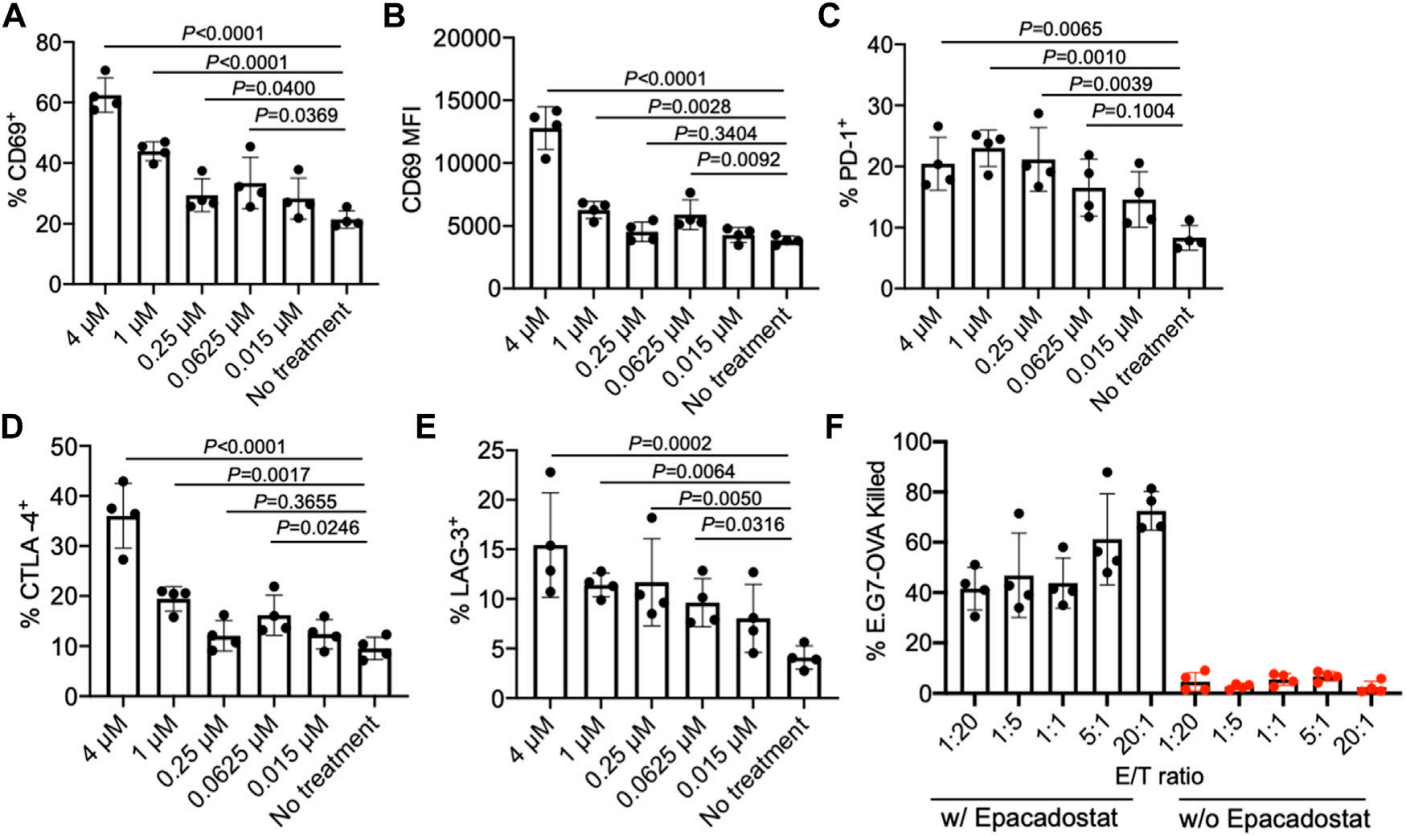
IDO1 inhibitor, epacadostat, can activate CD8^+^ T cells and improve killing of tumor cells. **(A)** Percentage and **(B)** MFI of CD69^+^ OT1 cells after treatment with different concentrations of epacadostat for 16 h. Also shown are the percentages of **(C)** PD-1^+^, **(D)** CTLA-4^+^, and **(E)** LAG-3^+^ OT1 cells after treatment with epacadostat for 16 h. **(F)** Percentage of killed E.G7-OVA cells by OT1 cells at different E/T ratios within 24 h in the presence or absence of epacadostat pretreatment. All the numerical data are presented as mean ± SD (0.01 < *p ≤ 0.05; **p ≤ 0.01; ***p ≤ 0.001).

We next studied the effect of epacadostat, an IDO1 inhibitor, on the activation status of CD8^+^ T cells. OT1 cells, a type of SIINFEKL-specific CD8^+^ T cells, were isolated from mice and cultured with different concentrations of epacadostat for 16 h. Compared to the untreated cells, OT1 cells treated with epacadostat showed an upregulated surface expression of CD69 (Figures 3A,B), indicating enhanced activation of OT1 cells after epacadostat treatment. Epacadostat-mediated activation of OT1 cells was concentration-dependent, with increased expression levels of CD69 at higher epacadostat concentrations (Figures 3A,B). The treatment of OT1 cells with epacadostat also resulted in the upregulated surface expression of PD-1, CTLA-4, and LAG-3 (Figures 3C–E, Supplementary Figures S2A–C), which is consistent with previous reports that more activated effector T cells tended to upregulate exhaustion markers. These experiments demonstrated that epacadostat-mediated inhibition of IDO1 can activate effector CD8^+^ T cells.

To study whether epacadostat-mediated enhanced activation of OT1 cells could impart an increased tumor-killing efficiency, we co-cultured epacadostat-pretreated OT1 cells with E.G7-OVA tumor cells for 24 h at an E/T ratio of 1/20, 1/5, 1/1, 5/1, and 20/1, respectively. OT1 cells without epacadostat pretreatment were used as controls. Strikingly, OT1 cells pretreated with epacadostat showed a significantly improved E.G7-OVA killing efficiency in comparison with the control OT1 cells without epacadostat pretreatment, with a ∼10-fold and ∼30-fold increase in killing efficiency at the E/T ratio of 1/20 and 20/1, respectively (Figure 3F). Even at an E/T ratio of 1/20, epacadostat-pretreated OT1 cells showed a ∼10-fold E.G7-OVA killing efficiency compared to control OT1 cells at an E/T ratio of 20/1 (Figure 3F). These experiments demonstrated that epacadostat-mediated IDO1 inhibition can significantly improve the activation status and tumor-killing capability of effector CD8^+^ T cells.

### Effect of Epacadostat on DCs

**FIGURE 4:**
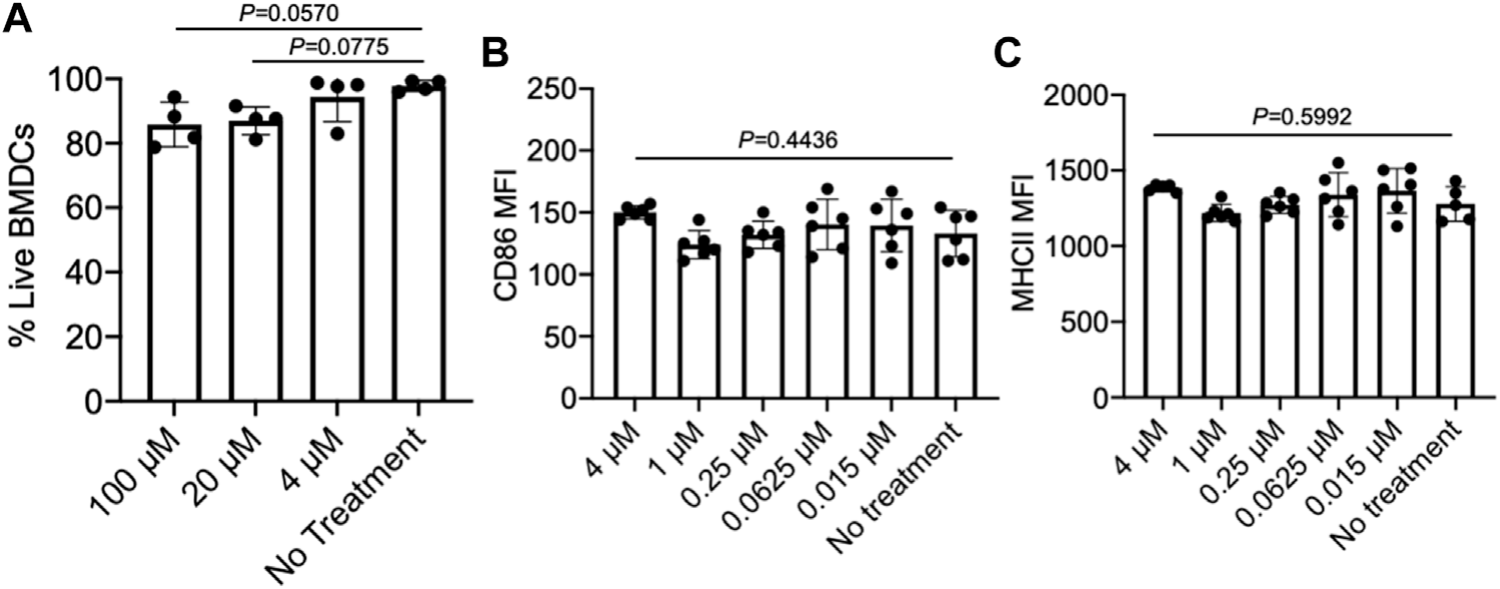
Epacadostat shows a minimal effect on the survival and activation status of DCs. **(A)** Viability of BMDCs after treatment with different concentrations of epacadostat for 48 h. **(B)** CD86 MFI of BMDCs after treatment with different concentrations of epacadostat for 24 h. **(C)** MHCII MFI of BMDCs after treatment with different concentrations of epacadostat for 24 h. All the numerical data are presented as mean ± SD (0.01 < *p ≤ 0.05; **p ≤ 0.01; ***p ≤ 0.001). *In situ* DC recruitment of gels loaded with GM-CSF and epacadostat.

As we aim to develop a material system that can simultaneously recruit DCs and activate tumor-infiltrating CD8^+^ T cells, we also examined the effect of epacadostat on the viability and activation status of DCs. We first tested the cytotoxicity of epacadostat against bone marrow-derived DCs (BMDCs) by analyzing the viability of BMDCs after 48 h co-culture. As a result, BMDCs treated with epacadostat showed similar viability to control BMDCs without epacadostat treatment at an epacadostat concentration of up to 100 µM (Figure 4A), demonstrating the minimal cytotoxicity of epacadostat towards BMDCs. We then studied whether epacadostat treatment would affect the activation status of BMDCs by examining the expression level of CD86 and MHCII, two activation markers of DCs, via FACS assay. For all the tested concentrations (0.015, 0.0625, 0.25, 1, or 4 µM), epacadostat treatment resulted in a negligible change in the expression level of CD86 or MHCII (Figures 4B,C). These experiments confirmed the minimal effect of epacadostat on the viability and activation status of DCs.

**FIGURE 5:**
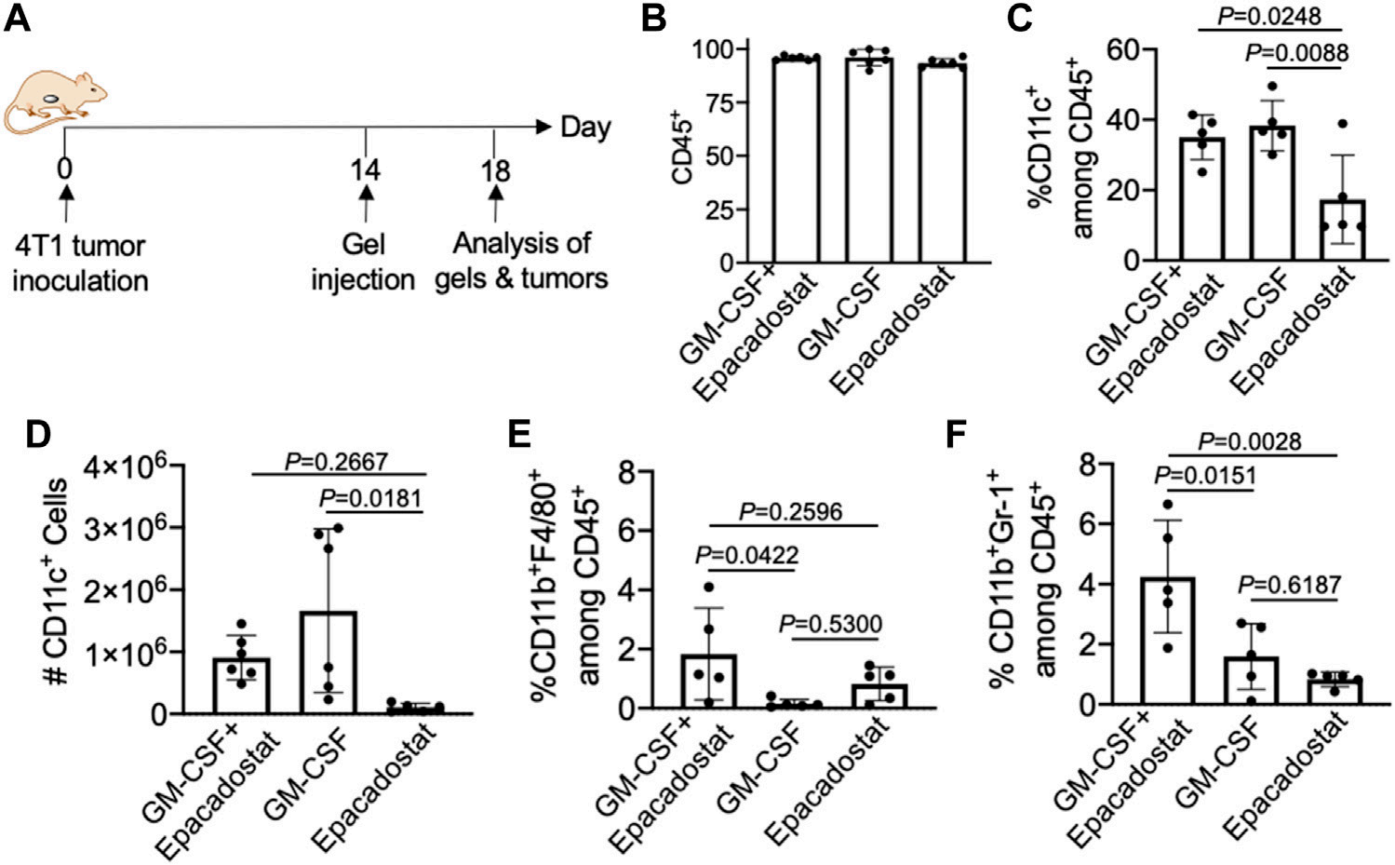
Gels loaded with GM-CSF and epacadostat can recruit DCs *in situ*. **(A)** Timeframe of the *in vivo* study. Gels encapsulating GM-CSF and epacadostat, gels encapsulating GM-CSF alone, or gels encapsulating epacadostat alone were injected adjacent to subcutaneous 4T1 tumors. **(B)** Percentage of CD45^+^ cells among the recruited cells in gels. **(C)** Percentage of CD11c^+^ DCs among CD45^+^ cells in gels. **(D)** Numbers of CD11c^+^ DCs in gels. **(E)** Percentage of CD11b^+^F4/80^+^ cells among the recruited cells in gels. **(F)** Percentage of CD11b^+^Gr1^+^ cells among the recruited cells in gels. All the numerical data are presented as mean ± SD (0.01 < *p ≤ 0.05; **p ≤ 0.01; ***p ≤ 0.001).

We next studied whether pore-forming gels loaded with GM-CSF and epacadostat, upon peritumoral injection, can recruit DCs *in situ* (Figure 5A). 4T1 tumor was established in Balb/c mice via subcutaneous injection of 4T1 cells into the flank. On day 14 when the tumors grew to a diameter of ∼6–8 mm, pore-forming gels encapsulating GM-CSF and epacadostat were injected adjacent to the tumors via an 18-gauge needle. Gels encapsulating GM-CSF alone or epacadostat alone were used as controls. After 4 days, gels were harvested for immune cell analysis. It is noteworthy that gels remained intact at the time of retrieval (Supplementary Figure S3A). Cells were successfully recruited to the gel site (Supplementary Figure S3B). The vast majority of cells present in the gels were CD45^+^ (Figure 5B), indicating the successful attraction of immune cells. Compared to gels encapsulating epacadostat alone, gels encapsulating GM-CSF and epacadostat or gels encapsulating GM-CSF alone recruited a significantly higher percentage of CD11c^+^ DCs among the recruited cells (Figure 5C). The total number of recruited DCs being recruited into the gel was also significantly higher in gels containing GM-CSF in comparison with gels containing epacadostat alone (Figure 5D), demonstrating GM-CSF-mediated recruitment of DCs. In addition to CD11c^+^ DCs, gels also recruited a small amount of CD11b^+^F4/80^+^ macrophages and CD11b^+^Gr1^+^ neutrophils (Figures 5E,F). A higher fraction of CD11b^+^F4/80^+^ macrophages and CD11b^+^Gr1^+^ neutrophils were detected in gels encapsulating GM-CSF and epacadostat, in comparison to gels loaded with GM-CSF alone or epacadostat alone (Figures 5E,F), indicating a potential effect of epacadostat on the immune cell homing process.

### Modulation of the Immunosuppressive Tumor Microenvironment

**FIGURE 6:**
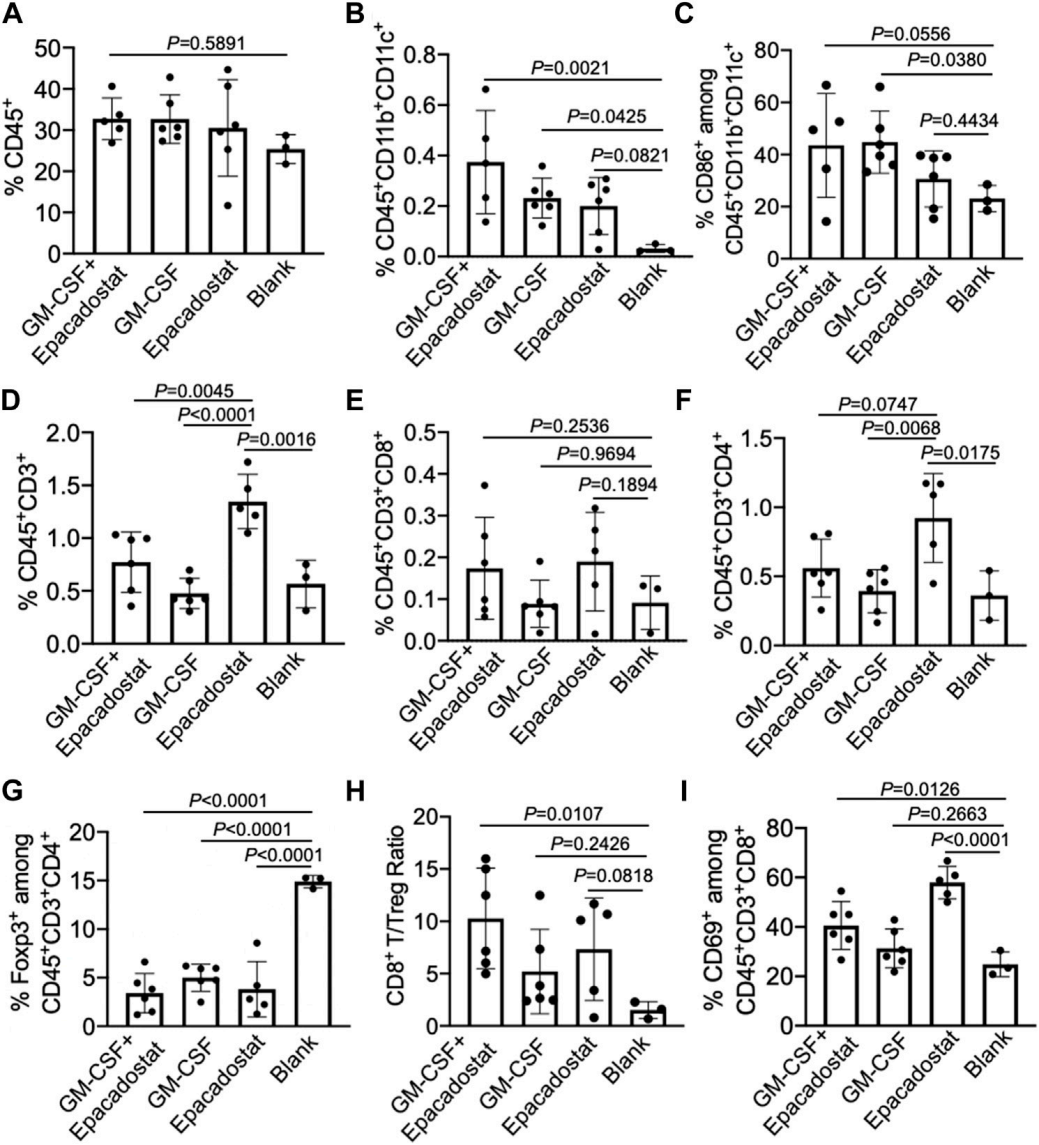
Gels loaded with GM-CSF and epacadostat can reshape 4T1 tumor microenvironment. 4T1 tumors were inoculated on day 0, followed by peritumoral injection of gel loaded with GM-CSF and epacadostat, gel loaded with GM-CSF alone, gel loaded with epacadostat, or blank gel on day 14. Gels and tumors were harvested for analysis on day 18. **(A)** Percentage of CD45^+^ cells in tumors. **(B)** Percentage of CD45^+^CD11b^+^CD11c^+^ DCs in tumors. **(C)** Percentage of CD86^+^ cells among CD45^+^CD11b^+^CD11c^+^ DCs in tumors. **(D)** Percentage of CD45^+^CD3^+^ T cells in tumors. **(E)** Percentage of CD8^+^ T cells in tumors. **(F)** Percentage of CD4^+^ T cells in tumors. **(G)** Percentage of FoxP3^+^ Treg cells in tumors. **(H)** CD8^+^ T/Treg number ratios in tumors. **(I)** Percentage of CD69^+^ cells among CD8^+^ T cells in tumors. All the numerical data are presented as mean ± SD (0.01 < *p ≤ 0.05; **p ≤ 0.01; ***p ≤ 0.001).

After confirming the recruitment of DCs to chemokine-loaded macroporous gels, we next analyzed whether peritumoral injection of gels encapsulating GM-CSF and epacadostat could improve the infiltration of DCs into tumors. 4T1 tumor cells were harvested, stained, and analyzed *via* FACS assay 4 days post gel injection. As expected, gels encapsulating GM-CSF and epacadostat resulted in an increased number of CD11c^+^ DCs in the tumor tissues compared to blank gels, with a 12-fold increase (Figures 6A,B). It is noteworthy that gels loaded with epacadostat alone also increased the tumor infiltration of DCs compared to blank gels (Figure 6B), presumably as a result of epacadostat-mediated immunomodulatory effects within the tumor microenvironment. In addition to the total number of DCs, gels loaded with GM-CSF or epacadostat also upregulated the expression of CD86 on the surface of intratumoral DCs in comparison with the blank gels (Figure 6C). These experiments demonstrated that peritumorally injected gels loaded with GM-CSF and epacadostat can recruit high numbers of DCs *in situ* and increase the number and activation status of tumor-infiltrating DCs.

We also analyzed whether gel treatment could alter the frequency and activation status of CD8^+^ and CD4^+^ T cells in the tumor microenvironment. Surprisingly, gels loaded with epacadostat alone resulted in the highest number of intratumoral CD3^+^ T cells among all treatment groups (Figure 6D), indicating the potent immunomodulatory effect of epacadostat. A similar trend was observed for CD4^+^ T cells in the tumor tissues (Figure 6F), with a highest CD4^+^ T cell number in tumors treated with gels encapsulating epacadostat alone. The number of intratumoral CD8^+^ T cells showed negligible changes across different groups (Figure 6E). However, compared to control tumors, the frequency of Foxp3^+^CD4^+^ Treg cells showed a significant decrease in tumors treated with gels encapsulating GM-CSF and epacadostat, GM-CSF alone, or epacadostat alone (Figure 6G). As a result, the CD8^+^/Treg ratio showed an increase in tumors treated with gels encapsulating GM-CSF and epacadostat in comparison with the control tumors (Figure 6H), which is often correlated with better CTL response and antitumor efficacy. We also observed an upregulated expression of CD69, an activation marker for effector T cells, by tumor-infiltrating CD8^+^ T cells in mice treated with gels encapsulating GM-CSF and epacadostat (Figure 6I), substantiating the T cell-activation effect of epacadostat. The expression levels of PD-1, CTLA-4, and LAG3 by tumor-infiltrating CD8^+^ T cells exhibited negligible differences among different treatment groups (Supplementary Figures S4A–C). These data demonstrated that gels loaded with GM-CSF and epacadostat can enhance the frequency and activation status of tumor-infiltrating CD8^+^ T cells, decrease the number of intratumoral Treg cells, and increase the overall CD8^+^/Treg ratio. We also analyzed the potential changes in the frequency and phenotypes of tumor-associated macrophages as a result of the gel treatment, which showed a negligible change at 4 days post gel injection (Supplementary Figure S5).

## Conclusion

To conclude, we have developed a macroporous gel-based system for simultaneous *in situ* DC recruitment and T cell activation. The pore-forming alginate gels are injectable, enable controlled release of GM-CSF and epacadostat, and can form macroporous structures after injection into the body. Upon peritumoral injection, gels can release GM-CSF to recruit and home DCs, which resulted in an increased number and improved activation status of DCs in the tumor microenvironment. The controlled release of epacadostat from gels also enabled *in situ* activation of CD8^+^ T cells, which together with the DC-recruiting property of gels, resulted in the enhanced tumor infiltration of CD3^+^ T cells, reduced numbers of Treg cells, and increased CD8^+^/Treg ratios. The ability to reshape the immunosuppressive tumor microenvironment of 4T1 TNBC, as achieved by our macroporous gel system, holds great promise for improving the CTL response and antitumor efficacy against solid tumors. *In situ* immunotherapy also has great potential for clinical translation by integrating with surgery to better prevent the recurrence and metastasis of tumors.

## Methods

### Materials and Instrumentation

Sodium alginate, sodium periodate, sodium borohydride, and other reagents were purchased from Sigma-Aldrich (St. Louis, MO, United States) unless otherwise noted. Epacadostat was purchased from MedChemExpress (Monmouth Junction, NJ, United States). Recombinant murine GM-CSF was purchased from PeproTech, Inc. (Cranbury, NJ, United States). Primary antibodies used in this study, including fluorescein isothiocyanate (FITC)-conjugated anti-CD45 (Invitrogen), PE-conjugated anti-CD11b (Invitrogen), PE/Cy7-conjugated anti-CD11c (Invitrogen), APC-conjugated anti-CD86 (Invitrogen), Alexa Fluor 700-conjugated anti- Ly-6G/Ly-6C (Invitrogen), PerCP/Cy5.5-conjugated anti-F4/80 (Invitrogen), Alexa Fluor 700-conjugated anti-CD206 (Invitrogen), PE/Cy7-conjugated anti-CD163 (Invitrogen), PE/Cy5.5-conjugated anti-CD3-ε (Invitrogen), Alexa Fluor 700-conjugated anti-CD8-α (Invitrogen), PE/Cy7-conjugated anti-CD4 (Invitrogen), PE-conjugated anti-CD69 (Invitrogen), APC-conjugated anti-Foxp3 (Invitrogen), PE/Cy7-conjugated anti-PD-1 (Invitrogen), PE-conjugated anti-CTLA-4 (Invitrogen), APC-conjugated anti-LAG-3 (Invitrogen), and FITC-conjugated anti-MHCII (Invitrogen), were purchased from Thermo Fisher Scientific (Waltham, MA, United States). Fixable viability dye efluor780 was obtained from Thermo Fisher Scientific (Waltham, MA, United States). All antibodies were diluted according to the manufacturer’s recommendations. Mouse CD3^+^ T cell isolation kit, dynabeads, and LS separation columns were purchased from Miltenyi Biotec (Bergisch Gladbach, Germany). FACS analyses were collected on Attune NxT flow cytometers and analyzed on FCS Express v6 and v7. Statistical testing was performed using GraphPad Prism v8. Small compounds were run on the Shimadzu LC40 ultra high performance liquid chromatography. Mechanical tests were performed on an AR-G2 rheometer (TA Instruments, New Castle, DE, United States).

### Cell Line and Animals

The E.G7-OVA and 4T1 cell lines were purchased from American Type Culture Collection (Manassas, VA, United States). Cells were cultured in RPMI 1640 containing 10% FBS, and 100 units/mL Penicillin/streptomycin (with 50 μg/ml G418 for E.G7-OVA cells) at 37°C in 5% CO_2_ humidified air. Female BALB/c mice were purchased from the Jackson Laboratory (Bar Harbor, ME, United States). Feed and water were available *ad libitum*. Artificial light was provided in a 12 h/12 h cycle. All procedures involving animals were done in compliance with National Institutes of Health and Institutional guidelines with approval from the Institutional Animal Care and Use Committee at the University of Illinois at Urbana-Champaign.

### Preparation of GM-CSF and Epacadostat-Loaded Pore-Forming Alginate Gels

Alginate (20 mg/ml) was dissolved in PBS and loaded into Syringe #1. GM-CSF and epacadostat in PBS were loaded in Syringe #2. Porogen beads and CaSO_4_ (156 mg/ml) were loaded in Syringes #3 and #4, respectively. Sequential mixing of Syringes 1–4 was performed *via* a Luer lock connector. After rapid mixing, ∼100 μL gel was injected into the flank of each mouse.

### Pore Size Measurement

Macroporous hydrogels were immersed in PBS and placed under the optical microscope. At least 50 images were taken for different regions of the macroporous hydrogel. For each image, the pore size was measured with the assistance of ImageJ and averaged. These averaged numbers were then further averaged over at least 50 images to calculate the pore size of macroporous hydrogels.

### Porosity Measurement

Macroporous gels were placed in 1 ml of deionized water at ambient condition for a few hours. Gel wicking assay was performed using an absorbent Kimwipe (Kimberly-Clark) to touch only one side of the gel, allowing the water inside the pores to be drawn out by capillary force. The initial gel weight (W_i_) and the final gel weight after the wicking assay (W_f_) were measured. Porosity was calculated as 100%*(W_i_-W_f_)/W_i_.

### Mechanical Tests of Gels

The storage and loss moduli of pore-forming hydrogels were extracted from strain sweep tests at 1 Hz followed by a frequency sweep test at 0.5% strain under the ambient condition on an AR-G2 rheometer (TA Instruments). An 8 mm flat plate geometry equipped with a bottom Peltier plate on the instrument was used to test all the samples with the same procedure file. A loading gap of approximately 1,600 μm was used for each test. Any kind of spillover while running the test was avoided by trimming the edges as needed. No pretreatment or stress was applied to any sample for any measurement.

### Release of Epacadostat From Pore-Forming Alginate Gels

Gels loaded with epacadostat were placed in PBS in a 24-well plate. At selected time points, 100 µl aliquots of the supernatant were sampled for quantification of the released epacadostat by HPLC. A concentration series of epacadostat were used for determining the standard curve.

### Testing of Immune Cell Activation by Epacadostat

For CD8^+^ T cells, OT1 cells were treated with different concentrations of epacadostat (4, 1, 0.25, 0.0625, and 0.015 μM, respectively) for 16 h. Cells were then centrifuged, washed, and stained with PE/Cy5.5-conjugated anti-CD3, Alexa Fluor 700-conjugated anti-CD8, PE-conjugated anti-CD69, PE/Cy7-conjugated anti-PD-1, PE-conjugated anti-CTLA-4, APC-conjugated anti-LAG-3, and fixable viability dye efluor780 for 20 min, prior to flow cytometry analysis. To study the effect of epacadostat on DCs, we cultured day-7 BMDCs with different concentrations of Epacadostat for 16 h. Cells were washed and stained with FITC-conjugated anti-MHCII, PE/Cy7-conjugated ant-CD11c, APC-conjugated anti-CD86, and fixable viability dye efluor780 for 20 min prior to FACS analysis.

### *In vitro* Tumor Killing Assay

OT1 cells were pretreated with epacadostat (100 ng/ml) or PBS for 24 h, and then co-cultured with E.G7-OVA cancer cells prestained with Calcein AM at different E/T ratios (20/1, 5/1, 1/1, 1/5, and 1/20, respectively). The viability of E.G7-OVA cancer cells was analyzed by quantifying the fluorescence signal in the supernatants on a plate reader.

### *In vivo* Tumor Modulation Study

Balb/c mice were divided into four groups: gels loaded with Epacadostat (90 ng/ml) + GM-CSF (1 μg/gel), gels loaded with GM-CSF (1 μg/gel) alone, gels loaded with Epacadostat (90 ng/ml) alone, and blank gels (*n* = 6). 4T1 cells in Hank’s buffered salt solution (HBSS) were subcutaneously injected into the upper right flank of Balb/c mice on day 0. On day 14, gels were freshly prepared and subcutaneously injected adjacent to the tumors. On day 18, gels and tumors were harvested. Tumors were disrupted using a syringe plunger to release cells. Cells were collected, washed, and stained for flow cytometry analysis. For the evaluation of overall immune cell populations, cells were stained with FITC-conjugated anti-CD45, PE-conjugated anti-CD11b, PE/Cy7-conjugated anti-CD11c, Alexa Fluor 700-conjugated anti-Ly-6G/Ly-6C, PerCP/Cy5.5-conjugated anti-F4/80, PE/Cy5.5-conjugated anti-CD3, Alexa Fluor 700-conjugated anti-CD8, PE/Cy7-conjugated anti-CD4, and APC-conjugated anti-Foxp3. For the activation analysis of immune cells, cells were stained with APC-conjugated anti-CD86, Alexa Fluor 700-conjugated anti-CD206, PE/Cy7-conjugated anti-CD163, PE-conjugated anti-CD69, PE/Cy7-conjugated anti-PD-1, PE-conjugated anti-CTLA-4, and APC-conjugated anti-LAG-3.

### Flow Cytometry Analysis of Gels

Gels were harvested from mice and disrupted using a syringe plunger, releasing contained cells. Cells were pelleted, washed, and stained for flow cytometry analysis. For DC analysis, cells were stained with FITC-conjugated anti-CD45, PE-conjugated anti-CD11b, PE/Cy7-conjugated anti-CD11c, and APC-conjugated anti-CD86 for 20 min. For neutrophil and macrophage analysis, cells were stained with FITC-conjugated anti-CD45, PE-conjugated anti-CD11b, Alexa Fluor 700-conjugated anti-Ly-6G/Ly-6C, PerCP/Cy5.5-conjugated anti-F4/80, APC-conjugated anti-CD86, Alexa Fluor 700-conjugated anti-CD206, and PE/Cy7-conjugated anti-CD163.

### Statistical Analyses

Statistical analysis was performed using GraphPad Prism v6 and v8. Sample variance was tested using the F test. For samples with equal variance, the significance between the groups was analyzed by a two-tailed student’s t-test. For samples with unequal variance, a two-tailed Welch’s *t*-test was performed. For multiple comparisons, a one-way analysis of variance (ANOVA) with post hoc Fisher’s LSD test was used. The results were deemed significant at 0.01 < *p ≤ 0.05, highly significant at 0.001 < **p ≤ 0.01, and extremely significant at ***p ≤ 0.001.

## Data Availability

The raw data supporting the conclusion of this article will be made available by the authors, without undue reservation.
