# Supplementary material for: In Situ Dendritic Cell Recruitment and T Cell Activation for Cancer Immunotherapy
Source: Front Pharmacol. 2022 Aug 23;13:954955. doi: 10.3389/fphar.2022.954955 (PMC9445184; doi:10.3389/fphar.2022.954955)
Supplement: Supplementary file 1 [file DataSheet1.pdf]

In Situ Dendritic Cell Recruitment and T Cell Activation for Cancer Immunotherapy

Joonsu Han[a,#], Rimsha Bhatta[a,#], Yusheng Liu, Yang Bo, Hua Wang[a,b,c,d,e,f,g]*

[a]Department of Materials Science and Engineering, University of Illinois at Urbana-Champaign, Urbana, IL 61801, USA. [b]Cancer Center at Illinois (CCIL), Urbana, IL 61801, USA. [c]Department of Bioengineering, University of Illinois at Urbana-Champaign, Urbana, IL 61801, USA. [d]Carle College of Medicine, University of Illinois at Urbana-Champaign, Urbana, IL 61801, USA. [e]Beckman Institute for Advanced Science and Technology, University of Illinois at Urbana-Champaign, Urbana, IL 61801, USA. [f]Materials Research Laboratory, University of Illinois at Urbana-Champaign, Urbana, IL 61801, USA. [g]Institute for Genomic Biology, University of Illinois at Urbana-Champaign, Urbana, IL 61801, USA.

[#]These authors contributed equally.

*correspondence should be addressed to huawang3@illinois.edu

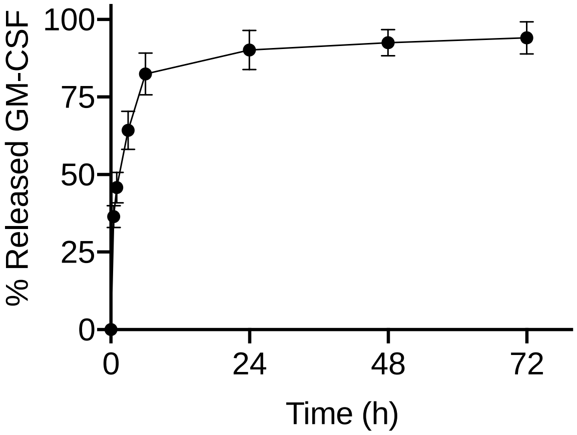

Figure S1. Release kinetics of GM-CSF from pore-forming alginate gels, as determined via the ELISA assay.

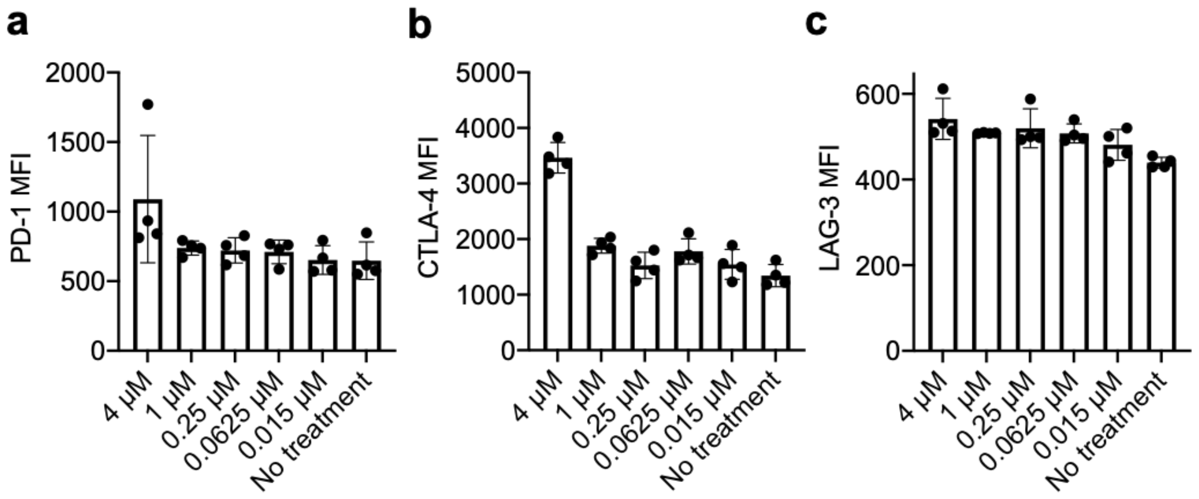

Figure S2. (a) PD-1, (b) CTLA-4, and (c) LAG-3 MFI of OT1 cells after treatment with epacadostat of varied concentrations for 24 h.

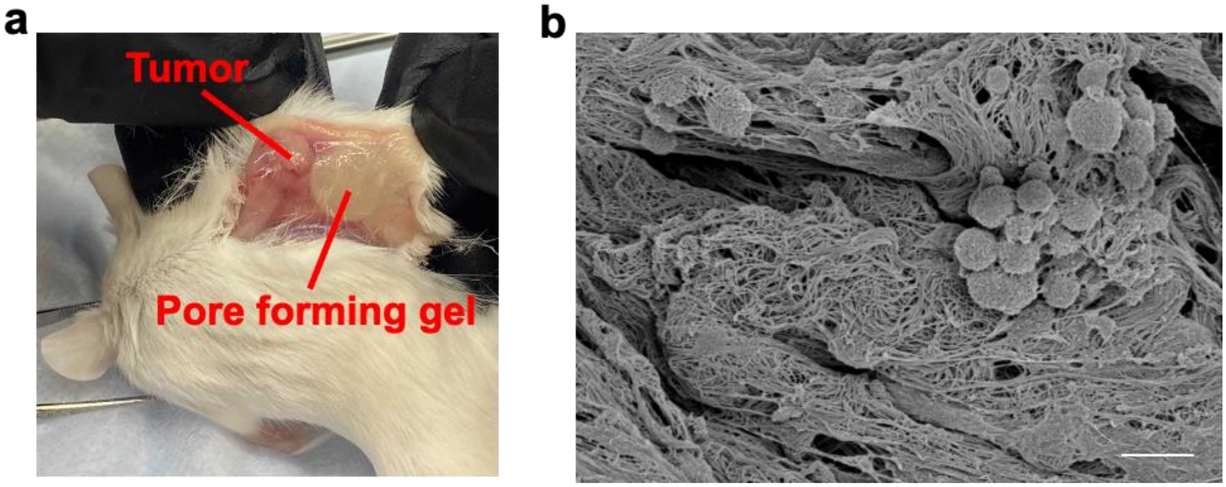

Figure S3. (a) Picture of pore-forming gels adjacent to the 4T1 tumor at 4 days post injection of gels. (b) Representative SEM image of GM-CSF-loaded pore-forming gels at 3 days post subcutaneous injection. Scale bar: 10 µm.

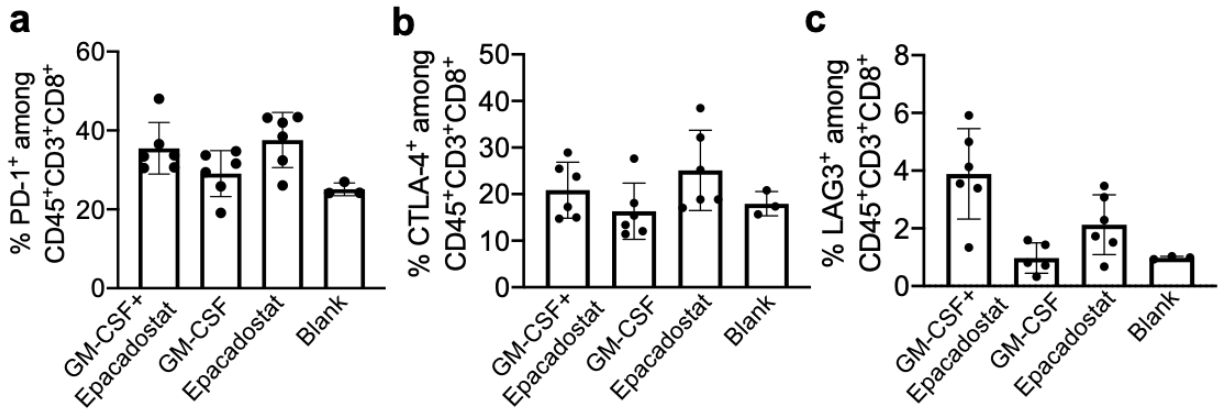

Figure S4. 4T1 tumors were inoculated on day 0, followed by peritumoral injection of gel loaded with GM-CSF and epacadostat, gel loaded with GM-CSF alone, gel loaded with epacadostat, or blank gel on day 14. Gels and tumors were harvested for analysis on day 18. Shown are the percentages of (a) PD-1^{+} cells, (b) CTLA-4^{+} cells, and (c) LAG-3^{+} cells among intratumoral $CD8^{+}$ T cells.

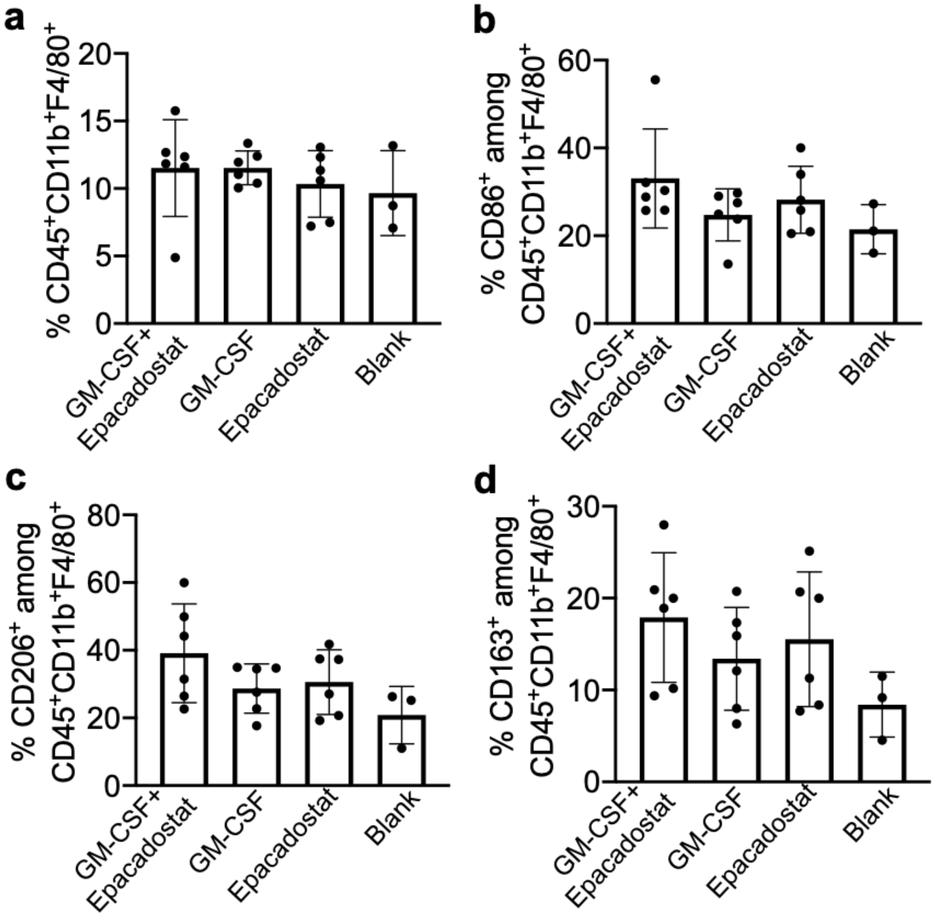

Figure S5. 4T1 tumors were inoculated on day 0, followed by peritumoral injection of gel loaded with GM-CSF and epacadostat, gel loaded with GM-CSF alone, gel loaded with epacadostat, or blank gel on day 14. Gels and tumors were harvested for analysis on day 18. Shown are (a) percentages of $CD45^+CD11b^+F4/80^+$ macrophages in tumors, (b) percentages of $CD86^+$ subpopulation among $CD45^+CD11b^+F4/80^+$ macrophages, (c) percentages of $CD206^+$ subpopulation among $CD45^+CD11b^+F4/80^+$ macrophages, and (d) percentages of $CD163^+$ subpopulation among $CD45^+CD11b^+F4/80^+$ macrophages.

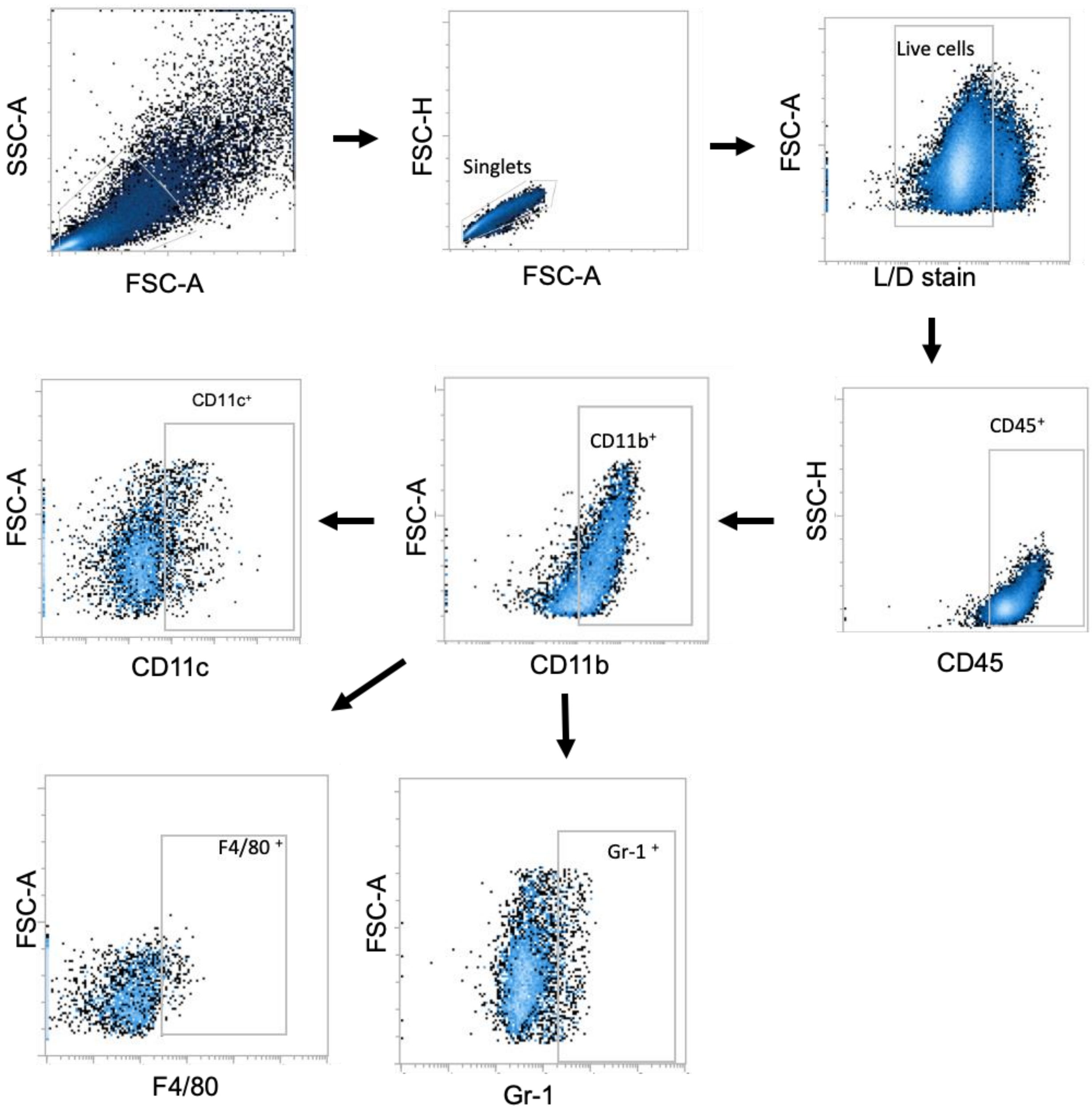

Figure S6. Representative gating strategy for gel samples.

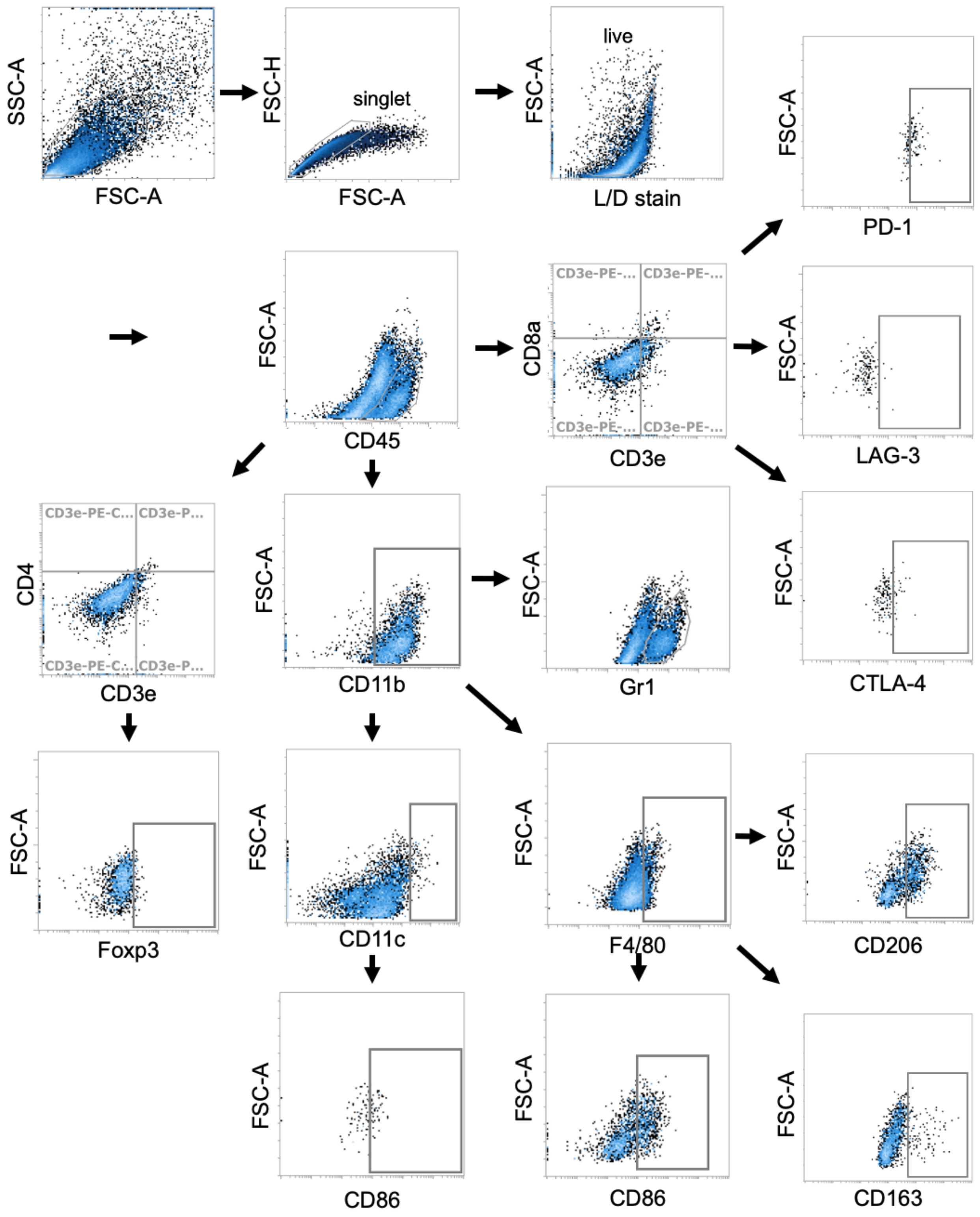

Figure S7. Representative gating strategy for tumor samples.
